# 3-Hydr­oxy-3-(methoxy­carbon­yl)penta­nedioic acid

**DOI:** 10.1107/S1600536809025598

**Published:** 2009-07-11

**Authors:** Lawal Aliyu, Nornisah Mohamed, Ching Kheng Quah, Hoong-Kun Fun

**Affiliations:** a School of Pharmaceutical Sciences, Universiti Sains Malaysia, 11800 USM, Penang, Malaysia; b X-ray Crystallography Unit, School of Physics, Universiti Sains Malaysia, 11800 USM, Penang, Malaysia

## Abstract

In the title compound, C_7_H_10_O_7_, the aliphatic chain is approximately planar [maximum deviation = 0.013 (1) Å] and makes a dihedral angle of 78.75 (7)° with the methoxy­carbonyl group. In the crystal, mol­ecules are linked *via* inter­molecular O—H⋯O and C—H⋯O hydrogen bonds into sheets parallel to (100). In the sheet, O—H⋯O hydrogen bonds generate *R*_2_^2^(9) and *R*_2_^2^(8) ring motifs.

## Related literature

For hydrogen-bond motifs, see: Bernstein *et al.* (1995 ▶). For bond-length data, see: Allen *et al.* (1987 ▶). For the stability of the temperature controller used for the data collection, see: Cosier & Glazer (1986 ▶).
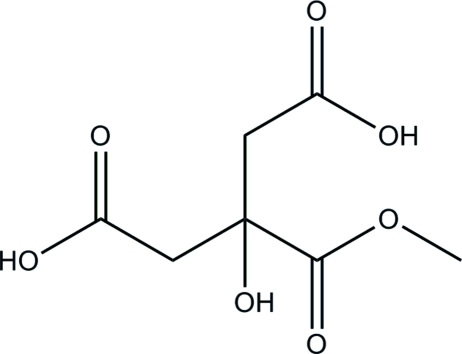


## Experimental

### 

#### Crystal data

C_7_H_10_O_7_
*M* *_r_* = 206.15
Orthorhombic, 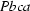

*a* = 12.7110 (4) Å
*b* = 5.8323 (2) Å
*c* = 23.8844 (7) Å
*V* = 1770.65 (10) Å^3^
*Z* = 8
Mo *K*α radiation
μ = 0.14 mm^−1^
*T* = 100 K
0.54 × 0.18 × 0.11 mm

#### Data collection

Bruker SMART APEXII CCD area-detector diffractometer
Absorption correction: multi-scan (**SADABS**; Bruker, 2005 ▶) *T*_min_ = 0.927, *T*_max_ = 0.985
54585 measured reflections
4404 independent reflections
4059 reflections with *I* > 2σ(*I*)
*R*_int_ = 0.030

#### Refinement

*R*[*F* ^2^ > 2σ(*F* ^2^)] = 0.041
*wR*(*F* ^2^) = 0.107
*S* = 1.12
4404 reflections
167 parameters
All H-atom parameters refined
Δρ_max_ = 0.56 e Å^−3^
Δρ_min_ = −0.25 e Å^−3^

### 

Data collection: *APEX2* (Bruker, 2005 ▶); cell refinement: *SAINT* (Bruker, 2005 ▶); data reduction: *SAINT*; program(s) used to solve structure: *SHELXTL* (Sheldrick, 2008 ▶); program(s) used to refine structure: *SHELXTL*; molecular graphics: *SHELXTL*; software used to prepare material for publication: *SHELXTL* and *PLATON* (Spek, 2009 ▶).

## Supplementary Material

Crystal structure: contains datablocks global, I. DOI: 10.1107/S1600536809025598/ci2844sup1.cif

Structure factors: contains datablocks I. DOI: 10.1107/S1600536809025598/ci2844Isup2.hkl

Additional supplementary materials:  crystallographic information; 3D view; checkCIF report

## Figures and Tables

**Table 1:** Hydrogen-bond geometry (Å, °)

*D*—H⋯*A*	*D*—H	H⋯*A*	*D*⋯*A*	*D*—H⋯*A*
O2—H1*O*2⋯O6^i^	0.84 (2)	1.85 (2)	2.6838 (8)	173 (2)
O3—H1*O*3⋯O1^ii^	0.87 (2)	2.01 (2)	2.8549 (8)	163 (2)
O4—H1*O*4⋯O5^iii^	0.91 (2)	1.73 (2)	2.6391 (9)	176 (2)
C4—H4*A*⋯O5^iv^	0.99 (1)	2.53 (1)	3.4035 (9)	147 (1)

Symmetry codes: (i) 
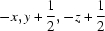
; (ii) 
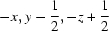
; (iii) 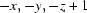; (iv) 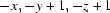.

**Fig. 1.**
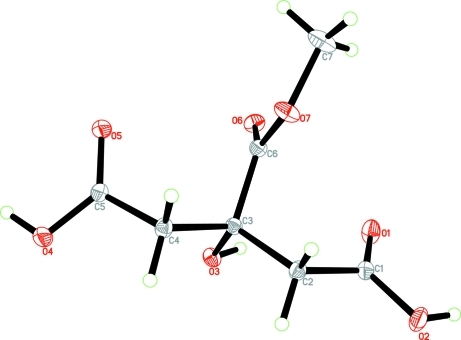
The molecular structure of the title compound, showing 50% probability displacement ellipsoids and the atom-numbering scheme.

**Fig. 2.**
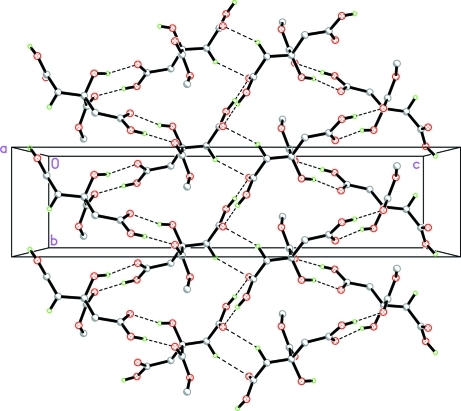
The crystal packing of the title compound, viewed along the a axis. H atoms not involved in hydrogen bonds (dashed lines) have been omitted for clarity.

## supplementary crystallographic information

### Comment

Research in natural products often results in the discovery of novel and interesting compounds. Chemical constituents from mango have been found to exhibit bioactivities against certain in silico disease models. Herein, we report the crystal structure of the title compound which was isolated from the mango extract.

The bond lengths (Allen *et al.*, 1987) and angles in the molecule (Fig. 1) are within normal ranges. Atoms C1, C2, C3 and C4 is approximately planar, with a maximum deviation of 0.013 (1) Å for atom C2. This plane makes a dihedral angle of 78.75 (4)° with the mean plane of methoxycarbonyl group (C6/C7/O6/O7).

In the solid state (Fig. 2), the molecules are linked *via* intermolecular O2—H1O2···O6 and O3—H1O3···O1 hydrogen bonds to generate *R*_2_^2^(9) ring motifs (Bernstein *et al.*, 1995) (Table 1) and pairs of intermolecular O4—H1O4···O5 hydrogen bonds form *R*_2_^2^(8) ring motifs. The crystal structure is further stabilized by intermolecular C4—H4A···O5 hydrogen bonds. The molecules are linked by these hydrogen bonds to form layers parallel to the (100).

### Experimental

The title compound was extracted by Soxhlet extraction method using methanol from oven-dried mangoes. The dried extract was dissolved in water and fractionated using different solvents. The ethyl acetate fraction was evaporated using rotary evaporator and the residue was purified using column chromatography (40% ethyl acetate: 60% n-hexane) to give crystals after washing with ethyl acetate. The crystals were later found to be suitable for *X*-ray analysis.

### Refinement

All H atoms were located in a difference Fourier map and refined freely.

### Crystal data

**Table:**

C_7_H_10_O_7_	*F*(000) = 864
*M**_r_* = 206.15	*D*_x_ = 1.547 Mg m^−^^3^
Orthorhombic, *P**b**c**a*	Mo *K*α radiation, λ = 0.71073 Å
Hall symbol: -P 2ac 2ab	Cell parameters from 9850 reflections
*a* = 12.7110 (4) Å	θ = 3.2–36.7°
*b* = 5.8323 (2) Å	µ = 0.14 mm^−^^1^
*c* = 23.8844 (7) Å	*T* = 100 K
*V* = 1770.65 (10) Å^3^	Plate, colourless
*Z* = 8	0.54 × 0.18 × 0.11 mm

### Data collection

**Table:**

Bruker SMART APEXII CCD area-detector diffractometer	4404 independent reflections
Radiation source: fine-focus sealed tube	4059 reflections with *I* > 2σ(*I*)
graphite	*R*_int_ = 0.030
φ and ω scans	θ_max_ = 36.7°, θ_min_ = 2.3°
Absorption correction: multi-scan (*SADABS*; Bruker, 2005)	*h* = −21→21
*T*_min_ = 0.927, *T*_max_ = 0.985	*k* = −8→9
54585 measured reflections	*l* = −39→39

### Refinement

**Table:**

Refinement on *F*^2^	Primary atom site location: structure-invariant direct methods
Least-squares matrix: full	Secondary atom site location: difference Fourier map
*R*[*F*^2^ > 2σ(*F*^2^)] = 0.041	Hydrogen site location: inferred from neighbouring sites
*wR*(*F*^2^) = 0.107	All H-atom parameters refined
*S* = 1.12	*w* = 1/[σ^2^(*F*_o_^2^) + (0.0517*P*)^2^ + 0.4849*P*] where *P* = (*F*_o_^2^ + 2*F*_c_^2^)/3
4404 reflections	(Δ/σ)_max_ = 0.001
167 parameters	Δρ_max_ = 0.56 e Å^−^^3^
0 restraints	Δρ_min_ = −0.24 e Å^−^^3^

### Special details

**Table:**

Experimental. The crystal was placed in the cold stream of an Oxford Cyrosystems Cobra open-flow nitrogen cryostat (Cosier & Glazer, 1986) operating at 100.0 (1) K.
Geometry. All esds (except the esd in the dihedral angle between two l.s. planes) are estimated using the full covariance matrix. The cell esds are taken into account individually in the estimation of esds in distances, angles and torsion angles; correlations between esds in cell parameters are only used when they are defined by crystal symmetry. An approximate (isotropic) treatment of cell esds is used for estimating esds involving l.s. planes.
Refinement. Refinement of F^2^ against ALL reflections. The weighted R-factor wR and goodness of fit S are based on F^2^, conventional R-factors R are based on F, with F set to zero for negative F^2^. The threshold expression of F^2^ > 2sigma(F^2^) is used only for calculating R-factors(gt) etc. and is not relevant to the choice of reflections for refinement. R-factors based on F^2^ are statistically about twice as large as those based on F, and R- factors based on ALL data will be even larger.

### Fractional atomic coordinates and isotropic or equivalent isotropic displacement parameters (Å^2^)

**Table:**

	*x*	*y*	*z*	*U*_iso_*/*U*_eq_	
O1	0.02576 (5)	0.61486 (11)	0.25826 (2)	0.01728 (11)	
O2	0.17885 (5)	0.80419 (12)	0.24936 (3)	0.01938 (12)	
O3	0.08405 (4)	0.23143 (9)	0.34206 (2)	0.01268 (10)	
O4	0.12975 (5)	0.07178 (11)	0.47512 (3)	0.01837 (12)	
O5	−0.02721 (4)	0.24507 (10)	0.46792 (2)	0.01487 (11)	
O6	−0.11179 (4)	0.42068 (10)	0.35333 (2)	0.01375 (10)	
O7	−0.04401 (4)	0.74583 (10)	0.38809 (3)	0.01586 (11)	
C1	0.10969 (5)	0.67852 (13)	0.27732 (3)	0.01260 (11)	
C2	0.14594 (5)	0.62879 (13)	0.33592 (3)	0.01235 (11)	
C3	0.07801 (5)	0.45077 (12)	0.36661 (3)	0.01026 (11)	
C4	0.12280 (5)	0.42501 (12)	0.42612 (3)	0.01211 (11)	
C5	0.06767 (5)	0.23834 (12)	0.45814 (3)	0.01218 (11)	
C6	−0.03676 (5)	0.53355 (12)	0.36864 (3)	0.01112 (11)	
C7	−0.14757 (6)	0.85003 (15)	0.38333 (4)	0.02113 (15)	
H2A	0.1457 (11)	0.771 (2)	0.3557 (6)	0.020 (3)*	
H2B	0.2200 (10)	0.580 (2)	0.3349 (6)	0.021 (3)*	
H4A	0.1116 (10)	0.567 (2)	0.4477 (6)	0.016 (3)*	
H4B	0.1959 (10)	0.390 (2)	0.4241 (5)	0.015 (3)*	
H7A	−0.1957 (12)	0.773 (3)	0.4079 (7)	0.033 (4)*	
H7B	−0.1406 (11)	1.010 (3)	0.3934 (6)	0.023 (3)*	
H7C	−0.1741 (14)	0.827 (3)	0.3455 (7)	0.039 (4)*	
H1O2	0.1539 (13)	0.834 (3)	0.2177 (7)	0.038 (4)*	
H1O3	0.0466 (12)	0.225 (3)	0.3117 (7)	0.032 (4)*	
H1O4	0.0934 (14)	−0.032 (4)	0.4959 (8)	0.046 (5)*	

### Atomic displacement parameters (Å^2^)

**Table:**

	*U*^11^	*U*^22^	*U*^33^	*U*^12^	*U*^13^	*U*^23^
O1	0.0175 (2)	0.0178 (3)	0.0166 (2)	−0.00488 (19)	−0.00472 (18)	0.00421 (19)
O2	0.0153 (2)	0.0256 (3)	0.0172 (3)	−0.0040 (2)	−0.00089 (18)	0.0107 (2)
O3	0.0149 (2)	0.0102 (2)	0.0130 (2)	0.00161 (16)	−0.00136 (16)	−0.00159 (16)
O4	0.0142 (2)	0.0180 (3)	0.0229 (3)	0.00341 (19)	0.00330 (19)	0.0089 (2)
O5	0.0123 (2)	0.0159 (2)	0.0164 (2)	0.00103 (17)	0.00198 (16)	0.00301 (18)
O6	0.0111 (2)	0.0156 (2)	0.0145 (2)	−0.00290 (17)	−0.00018 (16)	−0.00210 (17)
O7	0.0116 (2)	0.0115 (2)	0.0245 (3)	0.00166 (17)	−0.00251 (18)	−0.00385 (19)
C1	0.0128 (2)	0.0115 (3)	0.0135 (3)	0.0001 (2)	0.00038 (19)	0.0027 (2)
C2	0.0111 (2)	0.0132 (3)	0.0128 (3)	−0.0015 (2)	−0.00051 (19)	0.0029 (2)
C3	0.0101 (2)	0.0097 (2)	0.0110 (2)	−0.00015 (19)	−0.00016 (18)	0.00032 (19)
C4	0.0120 (2)	0.0133 (3)	0.0110 (3)	−0.0015 (2)	−0.00097 (19)	0.0013 (2)
C5	0.0129 (2)	0.0136 (3)	0.0100 (2)	0.0004 (2)	0.00019 (18)	0.0006 (2)
C6	0.0110 (2)	0.0106 (3)	0.0118 (3)	−0.00047 (19)	−0.00006 (18)	0.0002 (2)
C7	0.0146 (3)	0.0169 (3)	0.0319 (4)	0.0051 (3)	−0.0034 (3)	−0.0041 (3)

### Geometric parameters (Å, °)

**Table:**

O1—C1	1.2179 (9)	C2—C3	1.5365 (9)
O2—C1	1.3251 (9)	C2—H2A	0.956 (14)
O2—H1O2	0.838 (17)	C2—H2B	0.984 (13)
O3—C3	1.4094 (9)	C3—C6	1.5373 (9)
O3—H1O3	0.869 (16)	C3—C4	1.5386 (9)
O4—C5	1.3156 (9)	C4—C5	1.5037 (10)
O4—H1O4	0.91 (2)	C4—H4A	0.984 (13)
O5—C5	1.2290 (9)	C4—H4B	0.953 (13)
O6—C6	1.2152 (8)	C7—H7A	0.960 (16)
O7—C6	1.3256 (9)	C7—H7B	0.966 (15)
O7—C7	1.4543 (10)	C7—H7C	0.973 (17)
C1—C2	1.5020 (10)		
			
C1—O2—H1O2	108.5 (12)	C5—C4—C3	111.60 (6)
C3—O3—H1O3	111.0 (11)	C5—C4—H4A	106.0 (8)
C5—O4—H1O4	110.8 (12)	C3—C4—H4A	110.4 (8)
C6—O7—C7	115.21 (6)	C5—C4—H4B	108.9 (8)
O1—C1—O2	124.15 (7)	C3—C4—H4B	109.6 (8)
O1—C1—C2	123.93 (6)	H4A—C4—H4B	110.4 (11)
O2—C1—C2	111.90 (6)	O5—C5—O4	123.61 (7)
C1—C2—C3	113.75 (6)	O5—C5—C4	122.06 (6)
C1—C2—H2A	107.0 (8)	O4—C5—C4	114.32 (6)
C3—C2—H2A	110.5 (8)	O6—C6—O7	123.84 (6)
C1—C2—H2B	109.1 (8)	O6—C6—C3	124.41 (6)
C3—C2—H2B	110.7 (8)	O7—C6—C3	111.74 (6)
H2A—C2—H2B	105.5 (12)	O7—C7—H7A	109.5 (10)
O3—C3—C2	112.60 (5)	O7—C7—H7B	107.5 (8)
O3—C3—C6	110.48 (5)	H7A—C7—H7B	111.0 (13)
C2—C3—C6	109.64 (5)	O7—C7—H7C	109.2 (10)
O3—C3—C4	106.00 (5)	H7A—C7—H7C	106.4 (14)
C2—C3—C4	107.38 (5)	H7B—C7—H7C	113.3 (13)
C6—C3—C4	110.65 (5)		
			
O1—C1—C2—C3	11.29 (11)	C3—C4—C5—O4	120.28 (7)
O2—C1—C2—C3	−170.15 (6)	C7—O7—C6—O6	−7.76 (11)
C1—C2—C3—O3	65.49 (8)	C7—O7—C6—C3	170.92 (7)
C1—C2—C3—C6	−57.93 (8)	O3—C3—C6—O6	2.90 (9)
C1—C2—C3—C4	−178.20 (6)	C2—C3—C6—O6	127.56 (7)
O3—C3—C4—C5	−54.29 (7)	C4—C3—C6—O6	−114.18 (8)
C2—C3—C4—C5	−174.86 (6)	O3—C3—C6—O7	−175.77 (6)
C6—C3—C4—C5	65.51 (7)	C2—C3—C6—O7	−51.11 (8)
C3—C4—C5—O5	−60.67 (9)	C4—C3—C6—O7	67.15 (7)

### Hydrogen-bond geometry (Å, °)

**Table:**

*D*—H···*A*	*D*—H	H···*A*	*D*···*A*	*D*—H···*A*
O2—H1O2···O6^i^	0.84 (2)	1.85 (2)	2.6838 (8)	173 (2)
O3—H1O3···O1^ii^	0.87 (2)	2.01 (2)	2.8549 (8)	163 (2)
O4—H1O4···O5^iii^	0.91 (2)	1.73 (2)	2.6391 (9)	176 (2)
C4—H4A···O5^iv^	0.99 (1)	2.53 (1)	3.4035 (9)	147 (1)

Symmetry codes: (i) −*x*, *y*+1/2, −*z*+1/2; (ii) −*x*, *y*−1/2, −*z*+1/2; (iii) −*x*, −*y*, −*z*+1; (iv) −*x*, −*y*+1, −*z*+1.
